# Sitting less at work: a qualitative study of barriers and enablers in organisations of different size and sector

**DOI:** 10.1186/s12889-019-7148-8

**Published:** 2019-07-04

**Authors:** Kelly Mackenzie, Elizabeth Such, Paul Norman, Elizabeth Goyder

**Affiliations:** 1 School of Health and Related Research, University of Sheffield, Regent Street, Sheffield, S1 4DA UK; 2 Department of Psychology, University of Sheffield, Cathedral Court, 1 Vicar Lane, Sheffield, S1 2LT UK

**Keywords:** Sedentary behaviour, Sitting, Workplace, Occupation, Barriers and enablers, Focus groups, Qualitative, Organisational culture

## Abstract

**Background:**

Prolonged sitting is associated with a range of chronic health conditions and working in office-based jobs is an important contributor to total daily sitting time. Consequently, interventions to reduce workplace sitting have been developed and tested; however, no single intervention strategy consistently produces reductions in workplace sitting time. Exploring barriers and enablers to sitting less at work has been shown to support the development of more effective interventions. In order to address these barriers and enablers during the development and implementation of sit less at work interventions, it is important to understand how they may differ in different types of organisation, an area which has not yet been explored. The main aim of this study was to determine whether barriers and enablers to sitting less at work varied between organisations of different size and sector.

**Methods:**

A qualitative study design was used. Four organisations of different sizes and sectors participated: a small business, a charity, a local authority and a large corporation. A total of ten focus groups comprising 40 volunteer employees were conducted. Focus groups were audio-recorded and transcribed verbatim. Transcripts were then thematically analysed using pre-defined themes, but analysis also allowed for emergence of additional themes.

**Results:**

Barriers and enablers which were consistently raised by participants across all four organisations primarily included: individual-level factors such as habits and routines, and personal motivations and preferences; and factors relating to the internal physical environment. Barriers and enablers that differed by organisation mainly related to: organisational-level factors such as organisational culture, organisation size, and ways of working; and factors relating to the broader social, economic and political context such as the idea of presenteeism, and the impact of wider economic and political issues.

**Conclusions:**

This study found that although some themes were consistently raised by participants from organisations of different size and sector, participants from these organisations also experienced some different barriers and enablers to sitting less at work. For future research or practice, the study findings highlight that organisation-specific barriers and enablers need to be identified and addressed during the development and implementation of sit less at work interventions.

## Background

Prolonged sitting is now widely recognised as a risk factor for all-cause mortality [1] as well as many chronic health conditions including cardiovascular disease [2], metabolic syndrome [3], and low back pain [4]. Sitting at work has increased over recent decades due to the growth of office-based work and technological advances. For instance, observational studies have reported that office-based workers in England spend more than 60% of their total daily sitting time in work [5]. Prolonged sitting in the workplace has therefore become a public health concern.

Various interventions to reduce workplace sitting time have since been developed and tested including height-adjustable desks [6, 7], height-adjustable desks in combination with goal-setting, individual coaching and management involvement [8, 9], and low-cost behaviour change nudges such as emails from management, poster and computer prompts and social media [10]. Systematic reviews investigating the effectiveness of such interventions have demonstrated wide-ranging results, with no single intervention strategy, or even a range of strategies, producing consistent, long-term reductions in workplace sitting time [11–13]. Furthermore, a review by Gardner et al. [14] aimed to understand what the “active ingredients” of sedentary behaviour interventions may be and how they might best reduce sedentary behaviour. This review identified self-monitoring, problem solving, and restructuring the social or physical environment as the most promising behaviour change techniques, which could be used to inform future intervention design.

A recent qualitative systematic review [15] focused on factors important in the development, implementation and evaluation of sit less at work interventions. A key point highlighted in this review was the importance of understanding local barriers and enablers to sitting less at work as the first step in the development of sit less at work interventions. Identifying, and addressing barriers and enablers as part of the intervention development process is also consistent with guidance from the Medical Research Council [16] and supports the development of more effective interventions [15]. To date, only a small number of studies have reported barriers and enablers to workplace sitting interventions as a first step to intervention development [17–19]. More frequently, qualitative studies have explored barriers and enablers to sitting less at work as a standalone study, without explicitly stating that these findings will then be used to inform the development of interventions [20–22].

A further systematic review aimed to identify and synthesise qualitative evidence on factors that influenced feasibility and acceptability of sitting less at work [23]. Common barriers and facilitators to sitting less at work (not related to specific interventions) identified by this review were grouped using the ecological model of sedentary behaviour [24, 25] into: individual-level factors; work-related factors; organisational and social factors; and environmental factors. Individual-level barriers were identified as: sitting is a habit; and sitting less is an individual choice based on personal motivation. Work-related barriers included: the nature of the job; perceived loss of productivity; opportunities to move away from the desk was dependent on job role, e.g. those with management responsibilities or those with more autonomy over workload planning. Social and organisational-level barriers were reported as: the social norms of sitting; concerns about what colleagues may think or concerns about disturbing colleagues; and unsupportive workplace cultures. Finally, environmental-level barriers included: the use of furniture designed for sitting; and insufficient facilities to encourage incidental activity. In terms of enablers to sitting less at work, these primarily included organisational commitment or support, management permission to sit less, pleasant outdoor surroundings, good weather, and having centralised equipment away from individual desk space. This review synthesised findings from a variety of studies from different countries and from different types of organisation. The review reported that there was some evidence of differences in terms of cultural norms, where one study conducted in Singapore found that standing may be perceived as aggressive, but a study conducted in Sweden did not identify any cultural or social norms as a barrier to sitting less at work. However, this review did not explore whether there were any differences in perceived barriers and enablers based on organisation type, such as differences by sector and size, which is needed to fully understand how much of an influence such contextual factors may have on the effectiveness of sit less at work interventions.

A recent cross-sectional study of participants across 24 different worksites from academic, industrial and government sectors [26] found that there were both within and between workplace variations of objectively measured sedentary behaviour across the entire social ecological spectrum. However, this study only assessed a limited range of individual (job type and work engagement), cultural (lunch away from desk, walking at lunchtime, and face-to-face interaction), environmental (personal printer and office type) and organisational (sector) factors. In addition, the study was limited by the lack of contextual information collected. For example, regarding the cultural factors assessed, which related primarily to information on lunchtime and the opportunities this may bring to sit less at work, relevant contextual information such as where and when lunch is eaten, how long is the lunch break and whether there is a lunch break policy, was not collected. The inclusion of contextual information, obtained via exploring the perceptions of employees from a range of different types of organisations regarding the barriers and enablers to sitting less at work, would help to provide useful insights into factors that may influence workplace sitting behaviours in different organisations. Furthermore, this contextual information will be able to inform the real-world development and implementation of interventions to sit less at work [15]. Therefore, an in-depth exploration of factors that may present barriers or enablers to sitting less at work for different organisations and how organisational characteristics may influence these barriers and enablers is required.

The main aim of this study was to determine whether barriers and enablers to sitting less at work varied in organisations which differed in terms of size and sector. A secondary aim was to understand the barriers and enablers in each organisation, as a first step to developing sit less at work interventions.

## Methods

To address the aims of this study, a qualitative study design was adopted. A qualitative study design allowed for: an in-depth exploration of barriers and enablers to sitting less at work; the emergence of new themes or ideas that have not been previously identified in the literature; and an in-depth understanding of how these barriers and enablers differ by organisation and what the reasons for this may be [27].

### Organisations

This study aimed to recruit a variety of organisations of varying sector and type. This study purposefully recruited four organisations in Yorkshire, United Kingdom: a small-to-medium sized business (private sector); a charity (voluntary sector); a local authority (public sector and large); and a large corporation (private sector and large). The lead author recruited the small business opportunistically via a personal contact and the charity and local authority via professional contacts. The large corporation was suggested by a member of the research project’s public involvement panel. See Table 1 for a summary of organisation characteristics. None of the organisations had any formal sedentary behaviour initiatives in place, although the local authority had a bank of standing desks, which could be used as hot desks by any members of staff.

**Table 1:** Organisation characteristics

Characteristic	Small business	Charity	Local authority	Large corporation
**Total number of employees**	8	488	4146	119,300
**Sector**	Private	Voluntary	Public	Private
**Description of organisation**	Provides information technology support to a range of other businesses and comprises one Managing Director, two managers and five employees.	Manages homes, and provides care and support to vulnerable people. Departments include: Directors, Computer Services, Finance, Customer Services, Human Resources, Performance and Central Services, New Business, Housing Services, and Property Services.	Set in a Metropolitan Borough. Work covers four Directorates: Adults, Health and Wellbeing; Corporate Resources; Learning and Opportunities; and Regeneration and Environment.	Banking business. Only one branch participated. This branch had both business and corporate teams and comprises 25 staff and two team leaders.

### Participants

Convenience samples of participants in sedentary job roles were recruited from each of the four organisations via an email sent by a named contact within each organisation. In the small business, all eight employees received the recruitment email; in the charity and local authority, only office-based staff received the recruitment email; and in the large corporation only one branch of the business agreed to participate, which comprised 25 members of staff and two team leaders.

### Procedures

The lead author contacted staff who had expressed an interest in participating via email to arrange suitable times for the focus groups. Focus groups were conducted by the lead author (female, PhD student and Public Health Medicine Registrar) with participants at each organisation between January 2017–January 2018. Initial introductions established the lead author’s background in public health medicine and research interests. A focus group methodology was used to encourage dynamic idea generation amongst the group, leading to an in-depth discussion and the collection of rich data [28]. Focus groups were held during working hours in meeting rooms within the participants’ workplaces. The number of focus groups per organisation and number of participants in each focus group was based on participant availability to ensure we gained the maximum number of participants possible, rather than aiming for data saturation. This meant that in some instances, the number of participants per focus group was small. All participants provided written informed consent and ethical approval for this study was obtained from the School of Health and Related Research Ethics Committee at the University of Sheffield (ref no. 012219).

Participants completed a brief questionnaire providing some basic demographic (age bracket, gender, ethnicity, educational attainment) and work-related (job title, full time equivalent) details, and a self-reported estimation of percentage time spent sitting at work. Focus groups followed a topic guide (see Table 2), but were semi-structured to allow topics to be covered in a different order as appropriate, with additional follow-up or clarifications questions being asked when necessary. This topic guide was pilot-tested during the first focus group; no amendments were necessary. Focus groups lasted 30–60 min, were audio-recorded and transcribed verbatim.

**Table 2:** Focus group topic guide

Topic Covered	Template Questions
General perceptions about workplace sitting including its benefits and possible detrimental effects	a) What is your experience of/thoughts on workplace sitting? b) What is known, if anything, about the association between sitting and health? c) Do you suffer from any health effects of prolonged sitting?
Current barriers to reducing sitting time in the workplace	a) Are there any physical barriers e.g. disabilities/health problems/pregnancy? b) Are there issues regarding lack of understanding the importance of prolonged sitting on health? c) What are the norms in your office? Is it the norm to work standing or to sit less at work? What would your colleagues think? d) What is the management like? How may the organisation hinder you from sitting less? e) What is the environment that you work in like? Any barriers linked to this? f) Do you feel a need to remain seated? Is it something that concerns you?
Current enablers to reducing sitting time in the workplace	a) Are there any physical enablers that may encourage you to sit less e.g. musculoskeletal problems associated with prolonged sitting? b) Do you think having a good understanding of the importance of prolonged sitting in terms of health helps to reduce sitting time at work? c) What are the norms in your office? Do these help you to reduce your workplace sitting time e.g. supportive colleagues, others trying to reduce sitting? d) What is management support like? How does the organisation support you? e) What is the environment that you work in like? Are there any existing enablers linked to this e.g. centralised printers, green space? f) Do you feel a sense of motivation to sit less? Is it something that helps you?

### Analysis

Transcripts were uploaded onto NVivo Version 11 and a thematic analysis was undertaken. This was initially done using pre-defined themes which were taken from the findings from a recent qualitative systematic review [15]. These themes included: the nature of work, workload, time pressures, and individual working-style; feelings of self-consciousness or being a distraction to others; physical health effects, stress and impact on productivity; peer and/or management support and presence of social norms; the existing work environment; and the cost of an intervention. These were then re-named according to the data collected. Inductive thematic analysis was also carried out which allowed for the emergence of additional themes. From both the pre-defined and newly emergent themes, higher-order themes were determined forming a hierarchical structure.

The lead author read through transcripts multiple times to familiarise herself with the content prior to commencing the formal analysis. Data were coded by the lead author and from the codes hierarchical themes emerged. Direct quotations were used to describe the themes, enhancing credibility of the analysis. Data were firstly analysed as a whole dataset to determine the overall themes and sub-themes. These results were then broken-down by organisation to explore similarities and differences.

## Results

A total of 40 participants took part in 10 focus groups: two focus groups in the small business (*n* = 6); three focus groups in the charity (*n* = 15); three focus groups in the local authority (*n* = 14); and two focus groups in the large corporation (*n* = 5). Table 3 presents key participant characteristics by organisation. Participants’ job roles varied and included management, administration, technical support and professional roles.

**Table 3:** Participant characteristics (at July 2018)

Characteristic	Small business	Charity	Local authority	Large corporation	Total
Total number of participants	6	15	14	5	40
Total number of focus groups	2	3	3	3	10
Number of participants in each focus group	3, 3	4, 5, 6	4, 4, 6	3, 2	40
Total number of senior management-level participants	3	5	4	0	12
Mean age	31	38	41	43	38
Female (n)	1	9	10	4	24
Ethnicity					
- White British (n)	6	14	11	5	36
- Other (n)	0	1	3	0	4
Highest educational attainment					
- Degree or equivalent (n)	0	8	8	2	18
- Higher education (n)	1	1	3	0	5
- A level or equivalent (n)	3	1	1	1	6
- GCSEs grade A*-C or equivalent (n)	1	3	1	1	6
- No qualifications (n)	0	0	0	0	0
- Other (n)	1	2	1	1	5
Full-time (n)	5	13	13	4	35
Self-report % workday sitting time					
- 0–25% (n)	0	0	0	0	0
- 25–50% (n)	0	1	0	0	1
- 50–75% (n)	1	3	8	0	12
- 75–100% (n)	5	11	6	5	27

Table 3 above demonstrates the majority of participants in the focus groups were well-educated, white British females who work full-time and self-reported sitting at work for 75–100% of the time. However, it should be highlighted that there were some differences by organisation. For example, in the small business the majority of participants were younger males, and in the local authority and large corporation, the participants had a higher average age than the other organisations. Also, when comparing the number of participants in the focus groups to the total number of employees, the small business has a much higher proportion of participants involved in the workshops (6 out of 8) when compared to the other three organisations. Although convenience sampling was used and hence representativeness was not aimed for, the high proportion of staff from the small business participating in this study suggests that their views may be more representative than those from the other organisations.

The initial analysis of all transcripts identified barriers and enablers which encompassed 13 lower-order themes from which emerged four higher-order themes. These higher-order themes were identified as: individual factors; organisational factors; the internal physical environment; and the broader social, economic and political context. Themes are not intended to be mutually exclusive but cut across and between the different levels of influence. Table 4 highlights these higher-order themes and their associated lower-order themes by organisation.

**Table 4:** Summary of key themes and sub-themes by organisation

Themes	Sub-themes	Organisation			
		SB	C	LA	LC
1. Individual factors	1.1. Habits and routines	X	X	X	X
	1.2. Personal motivations and preferences	X	X	X	X
	1.3. Concerns about distracting colleagues		X	X	
2. Organisational factors	2.1. Nature of work	X	X	X	X
	2.2. Organisational culture*	X	X	X	X
	2.3. Organisation size	X			
	2.4. Ways of working		X	X	X
3. The internal physical environment	3.1. Building location, facilities and layout	X	X	X	X
	3.2. The workplace is designed for sitting	X	X	X	X
	3.3. Current equipment and furniture			X	
4. The broader social, economic and political context	4.1. Sitting is the social norm, standing is counter normative	X	X	X	X
	4.2. The idea of presenteeism		X	X	
	4.3. Economic and political issues			X	

*Note: SB* Small business*, C* Charity*, LA* Local authority*, LC* Large corporation

*X = sub-theme present*

*All organisations identified issues relating to organisation culture as presenting barriers and/or enablers, but within this theme there was a great deal of variation

Sub-analysis by organisation revealed some key similarities and differences in terms of the barriers and enablers to sitting less at work experienced by staff.

1. Individual factors

Two of the three individual-level sub-themes which influenced sitting behaviours were found to be consistent across the four organisations, namely: habits and routines; and personal motivations and preferences. The third sub-theme, concerns about distracting colleagues, varied by organisation.

1.1. Habits and routines

A variety of different habits and routines were felt to facilitate sitting less at work, as participants described habits that they currently adopt to be more active, such as walking to meet customers and making a drink or using the toilets on a different floor. One participant stated,

“And so now in my new team when I feel my concentration flags, so you just feel like you know that post-afternoon time. I always take that opportunity to take a drink. And even just use the toilets that are like the floor down or something like that. Basically, finding a way around all that. Well if I am taking a drink then actually just look out the window and take a few minutes where I can actually rest my head and then come back. I found having a drink is, and making it yourself is nice.” (Participant I, local authority).

1.2. Personal motivations and preferences

A variety of different motivators were reported to facilitate sitting less across the organisations. For example, musculoskeletal health was identified as a motivator for some participants, with sitting breaks being prompted by feeling stiff or sore. Also, experiencing benefits of going outside in the fresh air, as one participant explained, “Because when you do get to go outside and that fresh air hits you even just for a few minutes, it’s nice… Literally just blowing the cobwebs away from your brain.” (Participant A, large corporation). The “need” for a break was signified by waning productivity or an awareness of being sat for too long.

A further motivation which facilitated sitting less was taking productive or purposeful sitting breaks, such as breaking-out to chat with colleagues, which was seen to have additional benefits in terms of building relationships and improving efficiency, as one participant explained,

“I think being that bit more sociable activity with the person you tend to get a bit more of a rapport. So probably later on down the line they are a bit more than rather than hide away, they will come and see you. I think that works well yeah just going to see them I think.” (Participant H, charity).

However, the comfort and convenience of sitting was found to be a motivation for remaining seated and a barrier to being more active to the extent that one participant stated, “Because HR [Human Resources] are in the office, in like their own office, they were just, if you wanted to ask someone a question, you just shout across the office.” (Participant B, charity).

1.3. Concerns about distracting colleagues

This theme was a more prominent issue for the charity and local authority as a barrier to sitting less and being more active at work. This related to the perception that being more active could distract or disturb colleagues, as described by one participant,

“I think as well because so many staff sit in one building, that even when you are going round about to talk to someone, you could be stood for two minutes but you are surrounded by other people. You don’t want to get in their way or distract them. So to actually get up and move about a bit is kind of quite difficult.” (Participant F, charity).

2. Organisational factors

This theme provided the most variation by organisation. All associated sub-themes, except for the nature of work, were found to have important differences relating to barriers and enablers to sitting less at work.

2.1. Nature of work

The nature of work proved to be a significant barrier to sitting less for many participants. Within organisations, certain job roles were highlighted as being particularly sedentary such as call centre work and administration roles. In addition, certain tasks, which required intense concentration, promoted periods of prolonged sitting, “…sometimes you can just be so engrossed in what you are doing that you totally forget and you think oh I haven’t been to the toilet for a while.” (Participant F, large corporation) and another participant stated, “…if I’m on something that I need to focus on, then that would motivate me to sit at my desk and finish it and you know work through lunch or work past finishing time.” (Participant E, small business).

The nature of work was also perceived as providing limited opportunities to take sitting breaks or to be more active. For example, one participant stated,

“I think it’s really difficult when you work in a setting where you don’t have active, you don’t have lots of meetings or outings to the community or whatever. If you are sort of drawn in a project or a business support environment, how to encourage sort of people getting up and doing different things.” (Participant I, local authority).

2.2. Organisational culture

This sub-theme was an issue brought up by all organisations but encompassed many different aspects, each of which differed by organisation. Firstly, conflicts between the corporate line and what transmits down to staff was an issue that only appeared in the local authority. This was particularly in reference to encouraging staff to take breaks away from the computer screen as part of the Display Screen Equipment Regulations and was described by one participant, “And yet there is a message, take regular breaks from your desk. So there is that corporate message but it isn’t really embedded.” (Participant F, local authority).

Secondly, the perception that there needs to be a reason or excuse to move away from the desk was identified as a barrier to sitting less particularly by the charity participants. One participant stated that they, “Try and use an excuse to get up and move about a little bit.” (Participant A, charity) and another said, “You have got to have a reason for it. So you’ve got to be going to see someone or to do something.” (Participant G, charity). This links into the individual factor of a motivation for sitting less being taking a purposeful or productive break.

Thirdly, lack of time and workload pressures were also highlighted as significant barriers to sitting less at work predominantly by the large corporation and local authority participants. One participant described workload pressures as, “There’s never an end. There’s always so much on. It’s just like right you have to do this today. You just sort of sit there with your blockers on and do it.” (Participant B, large corporation). Another participant highlighted lack of time and the resulting pressures that this brings,

“And I know particularly just in my directorate alone, a lot of things are coming in short notice. So it’s like the pressure’s on you. You can’t plan in time to do you know a leisure thing here or an outing thing here because you are getting things landed on you last minute. And then it’s all just about sticking to your computer and get through it as quickly as possible.” (Participant J, local authority).

Finally, the role of managers was identified as a key barrier to sitting less and moving more at work by staff in the local authority. For instance, some participants reported the lack of management support or micro-management as a barrier to sitting less at work, as highlighted by one participant,

“I’ve actually had a couple of times when I’ve seen like either former colleagues or I can be actually talking about work with somebody like you say away from your desk. And if you are longer than you’ve kind of indicated that you might possibly be. Or if they thought you were just going to the toilet and you might have bumped into someone so oh while I’m here I’ll just catch up with you about you know. Your managers can say sometimes are you alright, where’ve you been? Or I’ve actually known a new manager come to the toilets to try and find me.” (Participant G, local authority).

However, in contrast, participants from the small business and large corporation reported that their managers were relaxed and flexible, this allowed them to use their time more flexibly and meant that they did not feel the same pressures in terms of being accountable for all of their work time, “We’ve got a really relaxed manager. Yeah if you’ve got something on in the morning personally and you are off as long as you get your work done.” (Participant C, large corporation).

2.3. Organisation size

Organisation size was only identified as an important organisational factor by the small business. The fact they were a small organisation was suggested to be an enabler as they would remind each other to sit less at work, “…once it’s on the radar we will be reminding each other.” (Participant A, small business). However, the lack of funding associated with being a small business meant that they are unable to afford larger-scale initiatives to improve their health and wellbeing, as explained by one participant,

“And I think group activity is healthy for a working environment but we are not a corporate, we are not an organisation that has enough cash to sort of splash out on a Segway day. You know what I mean?... It would be nice you know but we don’t have that kind of cash around which you know other outfits might do.” (Participant A, small business).

2.4. Way of working

This sub-theme relates to both the use of flexi-time and homeworking. The use of flexi-time was identified by participants from the local authority and charity as a barrier to sitting less at work. This is due to staff trying to build-up their time in order to get an additional day-off, as one participant describes, “people are trying to cut their dinner hour as short as possible to build time up or because of their working patterns and so on.” (Participant J, local authority). It was felt that this then puts pressure on individuals to work harder and take fewer breaks.

Homeworking was reported as an enabler to sitting less and moving more whilst working by some participants from the local authority and large corporation due to the increased flexibility and convenience that homeworking allows. Conversely, some participants from the local authority felt that they were more sedentary when working from home due to the ingrained stigma that when working from home you were perceived by colleagues to be “slacking”. To counter this, these participants felt that they had to be continually available when working from home, as one participant stated,

“But when you are at home you want to be available because if you move away from your table and someone phones you, that’s all that’s there to distract you. You are working from home. You should be available to take this phone call. So that’s where that stigma is all perception isn’t it? Someone’s not doing what they should be.” (Participant I, local authority).

3. The internal physical environment

Two of the three sub-themes relating to internal physical environment were found to be very consistent across the four organisations, namely, building location, facilities and layout, and the workplace is designed for sitting. The sub-theme, current equipment and furniture, was found to vary by organisation.

3.1. Building location, facilities and layout

The building location, facilities and layout presented both opportunities and limitations to sitting less at work within the four organisations. Although there were inevitable differences within this theme across organisations resulting in variations in the details of the discussion, this sub-theme as a whole was reported consistently by participants from all organisations. For example, some participants from the charity reported that, in their workplace, the location and state of the stairs was a barrier for using them, whereas local authority participants explained that, in their workplace, the stairs are central and you reach them before the lift, which promoted their use. A lack of office space and break-out areas was a perceived barrier for sitting less by participants from all organisations, due to the fact there was nowhere to go within the building when breaking away from the desk that would discourage sitting. For example, one participant stated, “there’s no breakout spaces where there is an opportunity to stand. Everywhere where you can break-out or meet is a chair-based sort of scenario.” (Participant E, local authority) and another stated, “There are no real informal meeting areas.” (Participant F, charity).

3.2. The workplace is designed for sitting

The idea that “we designed our workplace to be a sitting workspace” (Participant A, small business) was described by many participants. The whole way that the office space is set-up with desks, standard meeting tables with chairs, lack of break-out spaces all promotes sitting and discourages movement. For example, one participant stated, “So you’ve got a PC on your desk. Your phone’s on your desk. There’s a lot of anchors to keep you at your desk, whether you are drafting a report, working emails or...” (Participant B, charity).

3.3. Current equipment and furniture

Current equipment and furniture primarily related to the pre-existence of standing desks, which only the staff in the local authority had access too. On the surface, this presented itself as an opportunity to sit less at work. However, there were associated barriers to the use of these desks such as anxiety that they will “stand out”, the perception that using standing desks would be a distraction to colleagues, and practical issues with the desk, e.g. not having enough desk space and cabled incorrectly. As a result of these barriers, the standing desks were reported to be underused. One participant stated barriers to use of these standing desks involved,

“A number of things, first of all the desks are not within the teams you are in, so you’re out on your own. They are not connected to the phone network etc., so if you are there you can just work with your laptop or read documents. They are sat in a very open area, so anyone who goes across and uses them are in direct view, so there is, some people think well what are you doing using standing desks etc.” (Participant K, local authority).

4. The broader social, economic and political context

One sub-themes relating to the broader social, economic and political context was found to be consistently raised by all four organisations, namely, sitting is the norm and standing is counter normative. The other two related sub-themes, the idea of presenteeism and economic and political issues, were found to vary by organisation.

4.1. Sitting is the norm and standing is counter normative

The perception that sitting is the norm was identified as a barrier to sitting less at work by many participants from all four organisations, as one participant stated, “It’s normal to be sat at your desk.” (Participant A, charity). Furthermore, breaking that social norm by standing at work was also highlighted as problematic, as one participant described,

“You are breaking from the norm, aren’t you? If you stand up it’s even if you are just sitting at your desk not interacting with any other members of staff, it still seems a bit odd to like stand up and take a bit of a stretch just because nobody else is doing it you know.” (Participant B, small business).

Meetings were particularly described as a barrier to sitting less due to the additional pressures and expectations of social norms in this setting, “We’ll have meetings that you know might be in an office space sector where you will go and you will have a sit-down meeting because they are in that same sort of work environment.” (Participant F, local authority). Furthermore, the frequency and length of meetings in some of the organisations was an additional barrier,

“There’re a lot of meetings in this organisation. There’s an awful lot. I’ve never known anything like it really. There’s loads and loads of meetings which obviously generally you are sat at. We do have some where you are kind of walking around which is nice but usually you are kind of sat down.” (Participant J, charity).

4.2. The idea of presenteeism

The idea of presenteeism was described by participants as in order to be perceived as being productive, you need to be seen to be at your desk. This concept was perceived as a barrier to sitting less and moving more primarily by participants in the charity and local authority, and to a lesser degree in the large corporation. This theme did not emerge at all in the small business. One participant stated,

“It’s that presenteeism isn’t it? I guess just being seen. Especially I imagine, like I am in the team of there’s a bank of six desks and if someone’s gone you immediately know because you have to be aware because if someone calls then where they are and so they will state where they are. And thinking they’ve been off all day and you’ll look and maybe they’ve been in meetings. So it does seem like everyone has access to the calendars about you. You have I guess accountability to your team as well.” (Participant G, local authority).

4.3. Economic and political issues

Economic and political issues emerged as a barrier to sitting less at work in the local authority. This particularly referred to financial pressures facing local authorities which meant that initiatives primarily for enhancing employee wellbeing were not the priority. Furthermore, due to a pressure on managers to demonstrate that their staff are essential, sitting less and moving more is passively discouraged by the need to be sat at desks, which is also linked to the idea of presenteeism above. This was highlighted by one participant,

“I think for some managers it is all about the cuts being made. You know making sure their team are visible. And then that means that they’re essential because they are visible. Not necessarily but you know the productivity of the team or the need for that team is you know greater than visibly being somebody sat at their desk.” (Participant G, manager, local authority).

In addition, as the local authority is publicly funded, they are often under pressure due to external political factors. One participant described this,

“Yeah there’s a lot of pressure and financial you know pressures as well as culturally we are being scrutinised to a level that you know Grenfell fire [large tower block fire in the United Kingdom] and stuff not to bring that up but the councils are under pressure. This is public money that we are spending, so we need to be seen to be spending it correctly.” (Participant K, local authority).

## Discussion

The main aim of this study was to determine whether barriers and enablers to sitting less at work varied in organisations which differed in terms of size and sector. This study found that, although some themes were consistently raised by participants from organisations of different size and sector, participants from these organisations also experienced some differences which may be important to consider when developing and implementing sit less at work interventions.

Overall, four major influencing factors were identified: individual factors; organisational factors; the internal physical environment; and the broader social, economic and political context. These are consistent with the ecological model of sedentary behaviour [24, 25], which proposes that sedentary behaviours are influenced at multiples different levels, such as at an individual-, social-, community/organisational-, environmental-, and policy-level, which interact and feedback to each other as part of a dynamic system. Hadgraft et al. [23] recently conducted a qualitative systematic review which explored barriers and enablers to sitting less at work and also grouped emerging themes in line with the ecological model of sedentary behaviour [24, 25]. Furthermore, the sub-themes identified by this present study were broadly consistent with barriers and enablers identified by the review by Hadgraft et al. [23].

Sub-themes identified in this study which were consistently raised by participants from all four organisations, included: habits and routines; personal motivations and preferences; the nature of work; building location, facilities and layout; the fact that the workplace is designed for sitting; and the idea that sitting is the norm and standing is counter normative. These similarities mainly referred to individual factors and issues relating to the internal physical environment, which tend to be associated with the inevitable constraints of office work. These constraints were similar across all organisations as this study intended to assess barriers and enablers to sitting less at work in organisations where office-based work was predominant, so this finding is unsurprising. Furthermore, although sub-themes relating to the internal physical environment were broadly consistent across the four organisations, the actual environments were inevitably different for each organisation. These differences are not necessarily a reflection on the size or sector of the organisation, but rather of the availability of internal facilities and physical location of the building. One difference in the local authority related to barriers to using and accessing standing desks. The other participating organisations did not have access to standing desks, explaining why this theme was specific to the local authority. Many intervention studies have focused on standing desks or sit-stand desks as the “solution” to the sedentary behaviour in the workplace problem [6, 7, 29–31]. However, only barriers (not enablers) to using the standing desks were reported by staff in the local authority, which may indicate that such an intervention may not be appropriate in every organisation. The local authority demonstrated other competing and interacting influences that may have mitigated against the use of standing desks, for example the organisational culture impacted on their use as participants reported that they did not want to “stand out” or look foolish. This highlights how ingrained office behaviours are and how this may relate to professional identities and status.

There were some other notable differences between the four organisations which particularly related to organisational factors and the broader social, economic and political context and have not previously been reported in the occupational sedentary behaviour literature. The sub-theme of organisational culture, which is “considered to be a set of collective norms that govern the behaviour of people within the company” [32], encompassed a range of issues which differed by organisation. For example, participants from the local authority raised issues relating to conflicts between the corporate line i.e. positive messages to sit less and move more at an organisational-level/from Directors and what is transmitted down to staff via middle management, where staff felt pressure to be seen working at their desk. In addition, participants from the local authority reported negative impacts of the role of the manager through lack of support to sit less at work and through the need to account for their time at work. In contrast, participants from the small business and the large corporation reported the role of the manager as an enabler to sitting less at work. Furthermore, participants from the local authority and the large corporation identified lack of time and workload pressures as significant barriers to sitting less, whilst participants from the charity highlighted the need for a reason to move more as a barrier. Organisation size was only identified by the small business as an issue, where participants highlighted that for them, working in a small organisation was both an enabler in terms of peer support and a barrier in terms of a lack of funding for sit less at work initiatives. These issues may be dependent on the personalities of individual managers as peer support could be obtained from small supportive internal team structures within a large organisation, but equally there could be an ingrained management ethos related to the culture of an organisation which could influence people’s actions and behaviours [32]. Having a clear understanding of the various impacts the prevailing culture of an organisation may have on its staff is important as it will ensure some of these organisation-specific barriers and enablers are accounted for when developing and implementing sit less at work interventions.

The way of working was also shown to vary by organisation. The misuse of flexi-time was highlighted by the local authority and charity as a barrier to sitting less at work. Homeworking was described by the participants from the charity and local authority (where homeworking was an accepted practice) as an enabler due to the flexibility and convenience. However, some participants from the local authority also reported stigma associated with homeworking as a barrier to being more active when working from home. This is an important issue given the way in which the working environment is changing, with many organisations in the United Kingdom now offering flexible working options to their employees [33]. The idea of flexible working is to give employees some choice over how much, when and where they work in order to enhance work-life balance [33]. The suggestion that flexible working (which in this study included the use of flexi-time and working from home) could negatively impact daily sitting time is in line with a previous study by Olsen et al. [34]. This qualitative study of office-based workers in a financial services organisation in Brisbane, Australia found that flexible working increased sitting time due to increases in electronic communications, as a result of employees being unaware where their colleagues were (home or the office). However, this study did not report any stigma related to home-working, or staff using flexi-time in a way that it was not originally intended. The differences in the reasons for potentially increased occupational sitting time between the study by Olsen et al. and this present study may be due to differences in organisational culture of the organisations included in the two studies.

With the exception of the sub-theme, sitting is the norm and standing is counter normative, the impacts of the broader social, economic and political contextual factors were also found to vary by organisation. The idea of presenteeism was found to be a prominent barrier to sitting less for the local authority and charity. Presenteeism is a normative practice played out in organisations. However, it is influenced by broader social discourses about productivity and human value, but at an organisational level, could be influenced by the internal culture and expectations. The most commonly used definition of presenteeism comes from Johns [35], “showing up for work when one is ill”, but in this study, presenteeism purely related to the idea that sitting at your desk and being contactable is perceived as being “at work”. This also links in with the issues relating to flexi-time and homeworking where perceptions about building up flexi-time and stigma associated with homeworking appear to be underpinned by the broader social factor of presenteeism. Furthermore, economic and political issues were only raised by participants from the local authority. Being a publicly funded organisation in a context of continually limited funds and funding cuts and external political pressures, it was felt that staff needed to be visibly working to demonstrate that they are needed, hence making them more “deskbound” to conform to the organisation standards of behaviour. In addition, it was reported to be difficult to justify spending on sit less at work initiatives for staff. These impacts of the wider economic and political issues were not explicitly reported in the other organisations.

Reasons for all the differences between organisations identified by this study may, in part, be explained by differences in organisational cultures. Cultural factors which have been identified as potentially important for informing time staff spend sitting at work include details of local organisation processes and structures, e.g. organisation size, sector, structure, and the wider political and economic landscape [36]. The political and economic landscape seems to be particularly relevant to the experiences of participants in the local authority with a clear sense that as they are publicly funded and subject to high levels of scrutiny and accountability there is an additional pressure to be using this money wisely and appropriately on public services. This is a key factor to be aware of when developing sit less at work interventions in local authority organisations. Furthermore, organisation size seems to play a role in the small business and may contribute to the enablers which emerged here, being a close-knit team. As identified by Such and Mutrie [36], the various contextual domains are “mutually reinforcing”, i.e. wider political and economic factors may influence other organisational cultural domains such as organisational values, strategy, structures and operations. Therefore, addressing the issue of organisational culture to support interventions to sit less at work, could be potentially beneficial.

The current study has implications in both research and practice. This study’s finding that there are variations in barriers and enablers by organisation, suggests that a “one-size-fits-all” solution to sitting less at work is not suitable. Although it may be possible to develop a tool which incorporates the barriers and enablers identified in this study to support the future development of sit less at work interventions, this research suggests there needs to be a more bespoke and tailored approach. The findings from a qualitative review by Mackenzie et al. [15], which explored factors important for the implementation of sit less at work interventions, were translated into an operational framework to guide both researchers and practitioners in the development, implementation and evaluation of such interventions. As part of the intervention development process, the first step of this framework was identified as engaging with employees and managers in the target organisation to understand the barriers and enablers to sitting less. This framework highlights the importance of first gaining a clear understanding of both the nature and context of the target organisation’s barriers and enablers to sitting less at work, as the initial phase of the intervention development process. The framework also advocates for the use of a participatory approach when developing and implementing sit less at work interventions. A participatory approach could also be a key strategy to account for barriers and enablers that may be present within different organisations and support the development of strategies to account for these issues. Furthermore, the present study has found that it is important to consider social ecological factors, particularly focusing on organisational culture, and the impacts that the broader social, economic and political context may have on different organisations, both which appear to have the greatest propensity for variation between organisations.

There are a number of strengths to this study. This is the first qualitative study to explore and compare the barriers and enablers to workplace sitting in organisations of different sizes and in different economic sectors. The use of focus groups as the method of data collection was a strength as it enabled dynamic idea generation and in-depth discussions. Finally, including participants of a range of ages, educational attainment and job roles (including both staff and management) ensured that richer data were collected from a variety of different perspectives. The study offers further insight into barriers and enablers to sitting less at work in different organisational contexts, which should be considered when developing sit less at work interventions.

This study has some limitations that should be noted. First, with the exception of the small business, only relatively small samples were taken to reflect the views of each organisation. Due to the convenience-based sampling methods, it is possible that the participants who volunteered were those already engaged or keen to address the issue of prolonged sitting in the workplace. Therefore, some additional barriers and enablers may not have been captured. However, as this study aimed to understand existing barriers and enablers to sitting less and not views on sit less at work strategies, it was felt that participants would still be able to provide a good account of the present issues. Secondly, a common limitation to qualitative research is the lack of generalisability of the findings. Nonetheless, the findings across the organisations are broadly in line with previous studies of barriers and enablers to reduce workplace sitting [23].

## Conclusions

This study has demonstrated that there are some barriers and enablers that are consistent across organisations of different size and sector particularly in relation to individual and internal physical environmental factors, which appear to be primarily linked to the constraints of office-based work. However, there are some key differences between the four organisations that are important for both researchers and practitioners to be aware of when considering developing and implementing strategies to support staff to sit less at work. These differences mainly relate to organisational culture, organisation size, ways of working, the idea of presenteeism and the impact of the wider political and economic context. Public and voluntary sector organisations may face more constraints and bureaucracy due to higher levels of scrutiny and accountability compared to private sector organisations, which may have more freedom to innovate and make changes. If these issues are thoroughly considered as part of the development of sit less at work interventions, this could influence the intervention effectiveness and promote external validity. Based on the findings of this paper, developing an understanding of relevant organisational and wider contextual factors could be an important first step when considering developing sit less at work interventions.

## Data Availability

The datasets used and/or analysed during the current study are available from the corresponding author on reasonable request.
